# The Functional Organization of Neocortical Networks Investigated in Slices with Local Field Recordings and Laser Scanning Photostimulation

**DOI:** 10.1371/journal.pone.0132008

**Published:** 2015-07-02

**Authors:** Melissa A. Erlandson, Olivier J. Manzoni, Ingrid Bureau

**Affiliations:** 1 U901 INMED, INSERM, Marseille, France; 2 UMRS 901, Aix-Marseille University, Marseille, France; Instituto de Neurociencias de Alicante UMH-CSIC, SPAIN

## Abstract

The organization of cortical networks can be investigated functionally in brain slices. Laser scanning photostimulation (LSPS) with glutamate-uncaging allows for a rapid survey of all connections impinging on single cells recorded in patch-clamp. We sought to develop a variant of the method that would allow for a more exhaustive mapping of neuronal networks at every experiment. We found that the extracellular field recordings could be used to detect synaptic responses evoked by LSPS. One to two electrodes were placed in all six cortical layers of barrel cortex successively and maps were computed from the size of synaptic negative local field potentials. The field maps displayed a laminar organization similar to the one observed in maps computed from excitatory postsynaptic currents recorded in patch-clamp mode. Thus, LSPS combined with field recording is an interesting alternative to obtain for every animal tested a comprehensive map of the excitatory intracortical network.

## Introduction

The organization of neuronal networks has been the object of investigations since the onset of neuroscience research. Networks are dynamic, changing with development, learning, aging and with insults. Hence, their organization demands to be re-assessed in an ever increasing number of conditions. The functional mapping of sensory cortical networks has been principally approached using two methods. The first monitors the synaptic activity of single cells while searching for presynaptic partners, whereas the second delivers stimuli and tracks the propagation of synaptic signals often with fluorescence imaging. Exhaustive connectivity matrices with the highest degree of resolution have been generated thanks to studies based on single cell electrophysiological recordings where presynaptic partners were searched either with electrophysiological methods such as in paired recordings [1] or optically such as with UV-evoked glutamate uncaging [2]. Stimulating with light considerably speeds up the process of mapping. In a laser scanning phostimulation (LSPS) set-up, small groups of neurons (< 50) are sequentially stimulated at different sites which were patterned so as to generate a connectivity matrix [3]. Connections are indexed at sites where stimulation produced synaptic responses. This method has been used to map basic functional connectivity in sensory cortices [2,4,5,6,7], in motor and prefrontal cortex [8,9,10], in subcortical structures [11,12,13,14] and in the spinal cord [15]. Other studies mapped cortical connectivity in non-basal conditions such as in the context of brain development [16,17,18,19], pathologies [10,20,21,22] or after associative learning [23]. But these experiments remain arduous and often limited to the investigation of one or two layers because of technical constraints imposed by patch-clamp. The frustration is more pronounced in cases when mapping is the end-point of a lengthy and challenging process that began with the genesis of an animal model or with the conditioning of its behavior or when animals were allowed to age for several months. In addition to patch-clamp recording, extracellular electrophysiology is routinely done in cortex either in the intact brain or in slices [24,25]. Cortex is ideally suited to this simple recording method due to the abundance of pyramidal neurons and to the parallel alignment of their apical dendrite. In slices, local field potentials are generally evoked with electrical stimulations in order to screen pharmacological agents or for testing protocols inducing long-term plasticity. Here, we combined the relative ease of extracellular field recordings with the rapidity of LSPS to generate functional connectivity maps in barrel cortex.

Extracellular recordings were performed in layers of barrel cortex while projections were stimulated with LSPS. We found that UV-evoked glutamate uncaging generated local field potentials large enough to be detected in a set-up normally used for patch-clamp recordings. Direct and synaptic potentials were distinguished based on their latency to onset and on their differential sensitivity to blockers of synaptic transmission. Maps generated from evoked synaptic local field negativities displayed a laminar organization similar to that of excitatory circuits investigated with patch-clamp recordings. Along the horizontal axis, the columnar organization of inter-laminar projections was visible albeit with a smaller diameter. Hence, LSPS combined with extracellular recordings provides an interesting solution to quickly map the entire neocortex circuitry and readily identify projections that will merit further in-depth investigations.

## Materials and Methods

### Electrophysiological recordings

All experiments were performed according to INSERM ethics. This study and protocols were approved by the ethics committee of Ministère de l'enseignement supérieur et de la recherche, France, under the reference #00094.01. C57Bl6 males were between 6 and 7 week old. They received an intraperitoneal injection of a Ketamine/Xylazine mix (65 mg/kg, 6 mg/kg) and a cervical dislocation prior to decapitation. Across-row barrel cortex slices (300 μm thick) were prepared as described [26] in an ice-cold solution containing (in mM): 110 choline chloride, 25 NaHCO_3_, 25 D-glucose, 11.6 sodium ascorbate, 7 MgCl_2_, 3.1 sodium pyruvate, 2.5 KCl, 1.25 NaH_2_PO_4_, and 0.5 CaCl_2_. Slices were transferred to artificial cerebrospinal fluid (ACSF) containing (in mM): 127 NaCl, 25 NaHCO_3_, 25 D-glucose, 2.5 KCl, 1 MgCl_2_, 2 CaCl_2_, and 1.25 NaH_2_PO_4_, aerated with 95% O_2_ and 5% CO_2,_ first at 34°C for 15 minutes and then at room temperature prior to use. ACSF was complemented with (in mM): 0.2 MNI-caged glutamate (Tocris), 0.005 (±)-CPP (Sigma) an antagonist of NMDA receptors, 4 CaCl_2_ and 4 MgCl_2_ for LSPS mapping. 100 nM TTX (Tocris), a blocker of voltage-gated sodium channels, 100 nM LY379268 (Tocris), an agonist of group 2 metabotropic glutamate receptors (mGluR) or 5 mM gabazine, a blocker of GABA_A_ receptors, was added to the extracellular medium for pharmacological tests. Perfusion was recirculating except during the wash of LY379268. Recordings were performed at room temperature in the B, C or D whisker columns. Neuronal activity was recorded extracellularly or in patch-clamp. For extracellular recording, thin borosilicate electrodes with low resistance (0.5–1 MΩ) were filled with extracellular medium (see above) and lowered ~120 μm deep into the tissue. For patch, standard glass electrodes (4–6 MΩ) were filled with intracellular solution containing (in mM): 128 K-methylsulfate, 4 MgCl_2_, 10 HEPES, 1 EGTA, 4 Na_2_ATP, 0.4 Na_2_GTP, 10 Na-phosphocreatine, 3 ascorbic acid; pH 7.25. Recorded neurons were 60–90 μm deep. Slices illuminated with infra-red light were inspected with a 4× and a 60× objective in order to position the recording pipettes in distinct layers (L). L6 and L4 neurons had small cell bodies compared to neurons located in adjacent layers and L4 had barrel-like structures. L5A was a clear band below L4 and above a denser L5B. No clear demarcation was visible below L1 and down to L4. Hence, recordings from L2 or L3 were obtained by placing pipettes in the lower or upper half of the supragranular layer, respectively.

Traces of extracellular recordings were sampled at 10 kHz and filtered at 800 Hz (Multiclamp 700b, Molecular devices). 50/60 Hz noise and harmonics were removed with a noise eliminator (HumBug, Quest Scientific). Traces of whole-cell voltage-clamp recordings were sampled and filtered at 10 kHz. Focal photolysis of caged glutamate was accomplished with a 2 ms 20 mW pulse of a UV (355 nm) laser (DPSS Lasers Inc.) through a 0.16 NA 4 × objective (Olympus). The full optical pathway and scanning system are described in [27]. The stimulus pattern consisted of 256 positions on a 16 × 16 grid (75 μm spacing). The uncaging grid was centered vertically on a barrel or between two barrels. The 9^th^ and 10^th^ lines were above L4 and L5A, respectively. A picture of the slice with the overlying uncaging grid was saved for every pipette position. In experiments where consecutive maps were obtained at different locations, the position of the uncaging grid was kept the same, guided by landmarks and dark spot-like irregularities visible at the surface of the slice. The UV stimuli were presented once every 700 ms in a spatial order designed to avoid consecutive glutamate uncaging at neighboring sites [4]. Three to four maps with different sequential orders of UV stimulations were acquired for every pipette position. Custom software for instrument control and acquisition [3] was written in Matlab (MathWorks, Inc.).

### Data analysis

For extracellular recordings, traces were smoothed with a 2 ms sliding window. The four traces evoked at every uncaging site were averaged together to construct mean single-position maps. Two types of responses were evoked by glutamate-uncaging: direct or synaptic responses. Maps of direct evoked responses were constructed based on the measurement of the peak responses evoked in a 5 ms window starting with the UV stimulus onset for each location of photostimulation. Two maps of direct responses were generated: The first for negative evoked potentials, the second for positive evoked potentials. Synaptic input maps were based on the peak amplitude of negative potentials within a 50 ms time window starting 5 ms after the UV stimulus onset. For voltage-clamp recordings, the traces were smoothed with a 1.1 ms sliding window. Three synaptic input maps were computed and averaged for every pipette position based on the amplitude of synaptic responses averaged over a 50 ms time window starting 5 ms after the UV stimulus onset. Single-position maps acquired from same layers were then used to generate group-average maps. Interpolation was performed on mean input maps for display purposes only.

The mean distance of L4 synaptic inputs feeding into L2/3 was calculated as follows: Ʃ(synaptic input × lateral distance from the recording site)/Ʃ(synaptic input).

The excitation profile of excitatory L2/3, L4 and L5A neurons under glutamate uncaging was investigated in a previous study in which the same stimulating protocol and animals as of strain, age and sex were used [23]. Spikes were recorded in the single-cell loose-seal mode and glutamate was uncaged over an 8 x 8 grid (spacing, 50 μm; dimension 350 x 350 μm). Neurons fired a total of three (L4), four (L2/3) or six (L5A) action potentials (AP) over the entire grid. They fired a single AP in 84% to 98% of trials. AP were evoked when stimulating perisomatically, at a mean distance of ~ 40 μm (computed from Ʃ(AP × distance from the recording site)/Ʃ AP). A larger grid (8 x 16; spacing, 75 μm; dimensions, 525 x 1125 μm) was used for generating the excitation profile of L5B pyramidal cells in order to test whether AP could be evoked when glutamate was uncaged in the superficial layers. L5B pyramidal cells fired a total number of 2.5 ± 0.6 action potentials over the entire grid (1 spike per site at 88%; n = 11 cells). These were evoked at one to five perisomatic sites, at a mean horizontal distance of 43 ± 3 μm from the cell-body. The mean distance along the vertical axis was 44 ± 4 μm from the cell-body. Spikes were never evoked when glutamate was uncaged in L2-4. This indicates that depolarizations generated in the apical dendrites of L5B pyramidal cell were sub-threshold. Hence, local field potentials recorded in L5B when stimulating in L2/3 should be interpreted as synaptic events evoked at L2/3 to L5B connections. A shorter laser pulse (1 ms) was used in previous studies. Bureau and collaborators reported that excitation of L4 and L5A neurons were about two times lower then, whereas the excitation of L2/3 pyramidal cells was the same [20]. We found that a 2 ms pulse excited L5B pyramidal cells more reliably (5/11 did not fire with a 1 ms pulse). Remarkably, increasing the duration of the laser pulse did not increase the mean distance from the cell-body at which action potentials were evoked, indicating that the mapping resolutions were similar (see Fig 2 of [9] and the Methods of [20]).

Data presented are mean ± s.e.m. The statistical p values are from Mann-Whitney tests or Wilcoxon tests.

## Results

**Fig 1:**
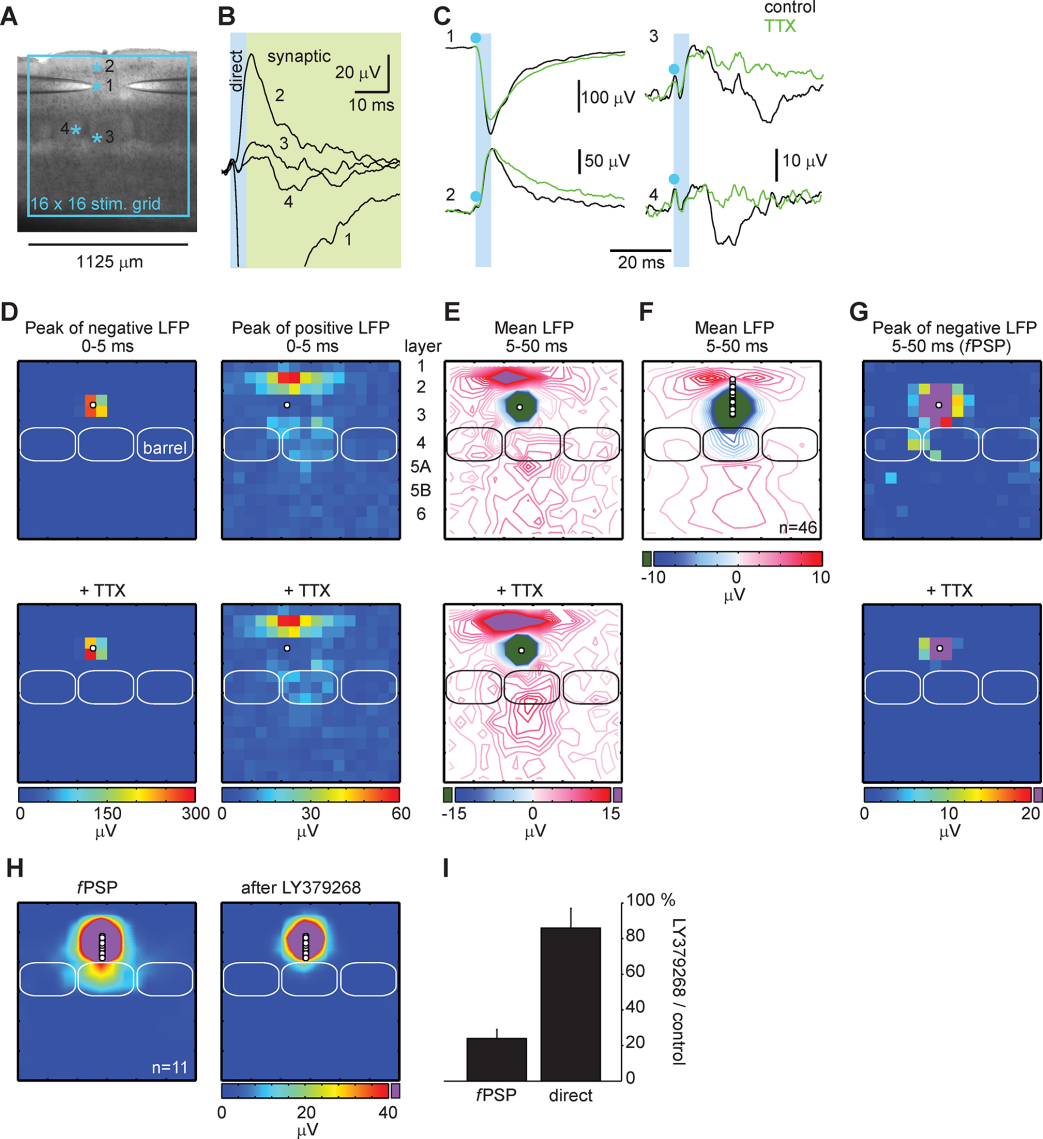
Field postsynaptic potentials (fPSP) are evoked by laser scanning photostimulation with glutamate uncaging in slice. **A**, One to two glass pipettes were placed in barrel cortex slice to record local field potentials. Responses where evoked with glutamate uncaging at sites positioned in a 16 × 16 grid with a 75 μm spacing. **B**, Examples of evoked local field potentials. Direct and synaptic responses could be distinguished based on their latency. The numbers refer to the stimulation sites shown in A. **C**, The black traces are the same as in B. Synaptic events were blocked by 100 nM TTX (in green). Blue dots indicate the stimulation onset and the light blue shade the time window for the rising of direct responses. **D**, Example of a map reporting with colors the peak amplitude of direct responses evoked 0 to 5 ms after the stimulus onset. Top panels are in control conditions, the bottom panels are in the presence of TTX. Left panels show the negative potentials, the right the positive potentials. The white circles indicate the position of the recording, the rectangles the position of barrels in the slice. All panels are from the same recording. **E**, Example of a map in control condition (top) and in the presence of TTX (bottom) showing the mean amplitude of local field potentials evoked 5 to 50 ms post stimulus onset. In blue, the isolines of regions with negative potentials; in red, the isolines of regions with positive potentials. The green and purple show regions where responses were larger than 15 μV. Same recording as in D. **F**, Group-average isoline map of recordings performed in layer 2/3. The isolines show the regions in the map where the mean amplitude of potentials evoked within the 5–50 ms time window were negative (blue) and positive (red). **G**, Example of a color map reporting the *peak* amplitude of negative potentials (fPSP map) evoked in control condition (top) and in the presence of TTX (bottom). Same recording as in D and E. **H**, Group-average map for recording performed in layer 3 prior (left) and after application of LY379268, an agonist of group 2 metabotropic glutamate receptors (right). **I**, The effect of LY379268 on synaptic events evoked by stimulations in layer 4 or on the direct responses evoked by stimulations in the vicinity of the recording pipette.

Extracellular field recordings and glutamate uncaging were first combined by Kötter and collaborators [25,28]. Recordings in each layer displayed a singularity in terms of inputs but neurons of deep layers were excited beyond threshold when superficial layers were stimulated [25], hampering a simple interpretation of the data in terms of interlaminar connectivity. In the present study, the experimental conditions were such that the detection of a synaptic response could unambiguously indicate the existence of direct connections between neurons with cell-bodies located at the stimulated site and the recorded neurons. Indeed, neurons recorded in the loose-seal mode fired solely when stimulated sites were located close to their cell-body: the half-width of the distributions of spikes as a function of distance from the soma was between 35 and 45 μm depending on layers (see Methods). Neurons fired most often a single spike. Could such stimulation generate local field potentials large enough to be detected from the extracellular space? We placed one to two low resistance glass pipettes for field recordings in the layer (L) 3 of barrel cortex and stimulated presynaptic neurons with glutamate uncaging following the pattern of a 16 × 16 sites grid (75 μm spacing; Fig 1A). Each site was stimulated four times with intervals of several minutes (see Methods) and the evoked responses were averaged on a pixel-by-pixel basis.

Like in LSPS combined with patch-clamp recordings in the voltage-clamp mode, glutamate-uncaging evoked both direct and synaptic events that could be detected from the extracellular space. Direct responses were measured in a short time window immediately following the stimulus onset (0–5 ms post stimulus). They were either positive or negative potentials (Fig 1B). Direct negative evoked potentials were up to several hundreds of μV and were resistant to TTX (100 nM) (93 ± 5% of control; n = 5 maps; Fig 1C and 1D). They were contained in a 3 × 4 pixel region in the close vicinity of the pipette tip (within 150 μm; Fig 1C and 1D). Thus, these negativities were analogous to the large depolarizations recorded in patch clamp when glutamate was uncaged on the cell-body or on close proximal dendrites [4]. Positive potentials measured in the same early time window were smaller but evoked at many more stimulation sites (Fig 1B–1D). They were also resistant to TTX (89 ± 4% of control; n = 5 maps; Fig 1D) suggesting they were return currents that "balanced" extracellular sinks evoked at a distance from the recording site [29] such as when glutamate was uncaged on apical dendrites (example: trace 2 in Fig 1C). Return currents were similarly observed when glutamate was uncaged below the recording site (example: trace 3 and 4 in Fig 1C) at greater distances, down to L4 and L5 (Fig 1D). These were most likely return currents of direct depolarizations evoked at the soma of deep pyramidal neurons whose apical dendrites extended close to the recording pipette.

Glutamate uncaged more than two pixels away (> 150 μm) from the recording pipette evoked post-synaptic events characterized by delayed onsets and small amplitudes (trace 3 and 4 in Fig 1C). However, traces in the 5–50 ms time window were complex such that when color maps displayed the averaged responses, both negative and positive responses emerged (shown as blue and red isolines respectively in Fig 1E). The group-average map generated from multiple recordings in L2/3 showed a spatial organization of these responses: one hotspot of negative responses occupied the barrel in L4 aligned with the recording pipettes and the L3 region directly above (Fig 1F). This pattern was reminiscent of maps computed from excitatory postsynaptic events (EPSC maps) recorded in single cell voltage-clamp mode displaying strong L4 to L2/3 projections. Hotspots of positive responses were visible above the recording sites and in layers below L4. Considering the long decay time of return currents of direct depolarization evoked in the presence of TTX (Fig 1C), it is most likely that the responses averaged over the 5–50 ms time window still integrated direct glutamate-evoked responses. Indeed, TTX did not block positive responses taken from the L1/2 and L5 regions but increased them slightly (+13 ± 3%; n = 85 responses over 5 maps; p < 0.001; Fig 1C and 1E). Hence, to optimize the detection of synaptic events, 'fPSP maps' were computed from the *negative peak* of field potentials detected in the 5–50 ms time window from then on (Fig 1G). Note how hotspots of synaptic responses appear at sites where they were masked by residual return currents when the mean amplitude was computed instead of the negative peak (compare the top panels in E and G).

fPSP measured at the peak were blocked by TTX (-93 ± 1%; n = 64 responses over 5 maps; p < 0.001; Fig 1G). A blocker of synaptic transmission that preserved the firing of presynaptic neurons was tested too. AMPA receptor antagonists were inappropriate because they blocked neuronal firing evoked by glutamate-uncaging. Neurotransmitter release at L4 stellate cell axon terminals is durably depressed by the activation of the presynaptic group 2 mGluR [30]. LY379268 (100 nM), a specific agonist of these receptors [31] had a major effect on fPSP: a 15 min bath application of LY379268 followed by a 20 min washout period decreased by -76 ± 5% (n = 11 maps; p < 0.001) the size of fPSP evoked by stimulations in L4 (Fig 1H and 1I). In contrast, direct negative potentials measured in the 0–5 ms time window were intact (86 ± 11% of control; NS), consistent with a presynaptic-only effect. These results validate our method for detecting synaptically-evoked local field potentials. We conclude that synaptic projections can be mapped with LSPS and extracellular recordings combined in slices.

We next investigated the map patterns as a function of cortical layers. Maps were obtained by moving pipettes from L6 to L2 according to landmarks in the slice. The position of the uncaging grid was kept constant with the L4/L5A border for reference (see Methods). Recordings were made simultaneously in two adjacent columns to increase the yield and a total of 122 single position maps were acquired out of 11 slices (n = 11 mice). Maps acquired from the two columns were re-aligned and pooled together in order to generate a functional connectivity map from an 'average column' for every layer.

**Fig 2:**
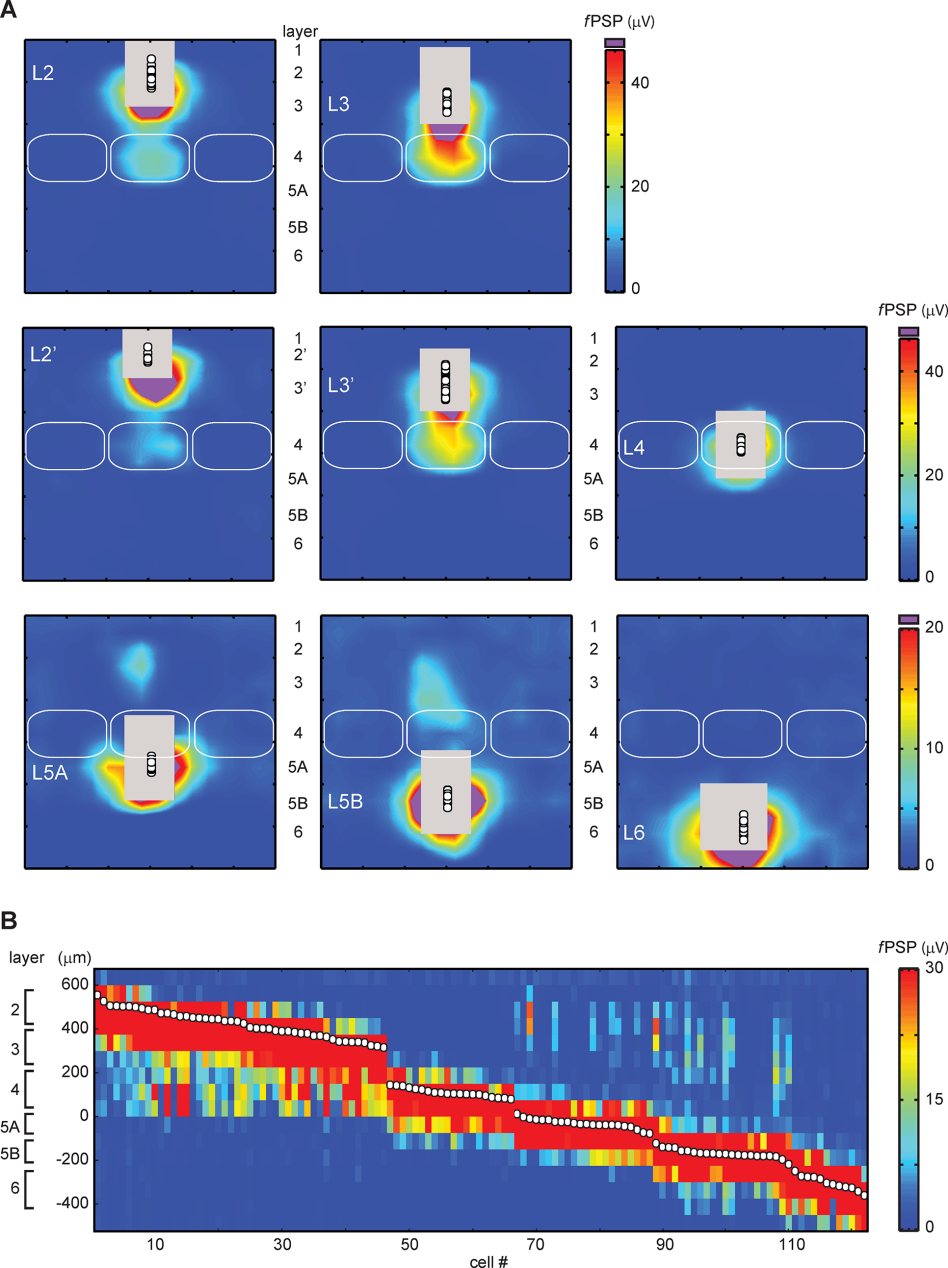
fPSP maps are generated for every layer of barrel cortex. **A**, Group-average maps for the layer 2, 3, 4, 5A, 5B and 6. L2 and L3 group-average maps were generated according to two conventions: In the upper row, L2 and L3 had similar thicknesses (n = 24 and 22 recordings, respectively). In the middle row, L2 was a narrow band (68 μm) below L1 and L3 was thicker (n = 10 and 36, respectively). L4, n = 20; L5A, n = 20; L5B, n = 21; L6, n = 12. Regions in purple are where fPSP peak amplitudes were greater than the upper limit of the color scale. Regions in grey are where directly-evoked responses prevented fPSP measurement. The solid white circles indicate the recording positions. **B**, Stack of maps for 122 recordings each compressed in one dimension.

Layers received unique patterns of projections: they all received their strongest inputs from themselves but also received weaker inputs from layers either deeper when they were in L2/3 or L4 or more superficial when they were in L5 or L6 (Fig 2A). This switch from ascending to descending projections around L4 was most obvious when looking at maps collapsed along the horizontal axis (Fig 2B).

There is no clear cytoarchitectural demarcation between L2 and L3. Previous studies have set the L2/3 border at midrange of the supragranular layer [27] or, more recently, few tens of microns below L1 leaving L2 as a narrow band [1,9,32]. Fig 2A presents the fPSP maps generated according to the two conventions. Both representations show that neurons close to barrels received stronger L4 inputs than neurons located more superficially. The collapsed representation of maps in Fig 2B indicates that the strength of L4 projections to superficial layers changed in a continuum. Another example of smooth transition was between L5A and L5B, despite the existence of a clear optical demarcation here. Indeed, L5A and L5B both received inputs from L2/3 but the center of mass slowly shifted downward for pipettes positioned deeper and deeper. In contrast, changes of map pattern were abrupt between L4 and L5A and between L5B and L6. Indeed, L4 received inputs from L5A only whereas L5A cells received inputs from L2/3, L5B and the bottom of L4. The L5B/ L6 switch occurred at 200 μm below the L4/L5A border and L6 cells received little inputs from L5B but none from L3 or L4. These features made the fPSP maps remarkably similar to the maps computed from excitatory post-synaptic current (EPSC) measurements obtained in single cell mode [7,9,23,33,34].

**Fig 3:**
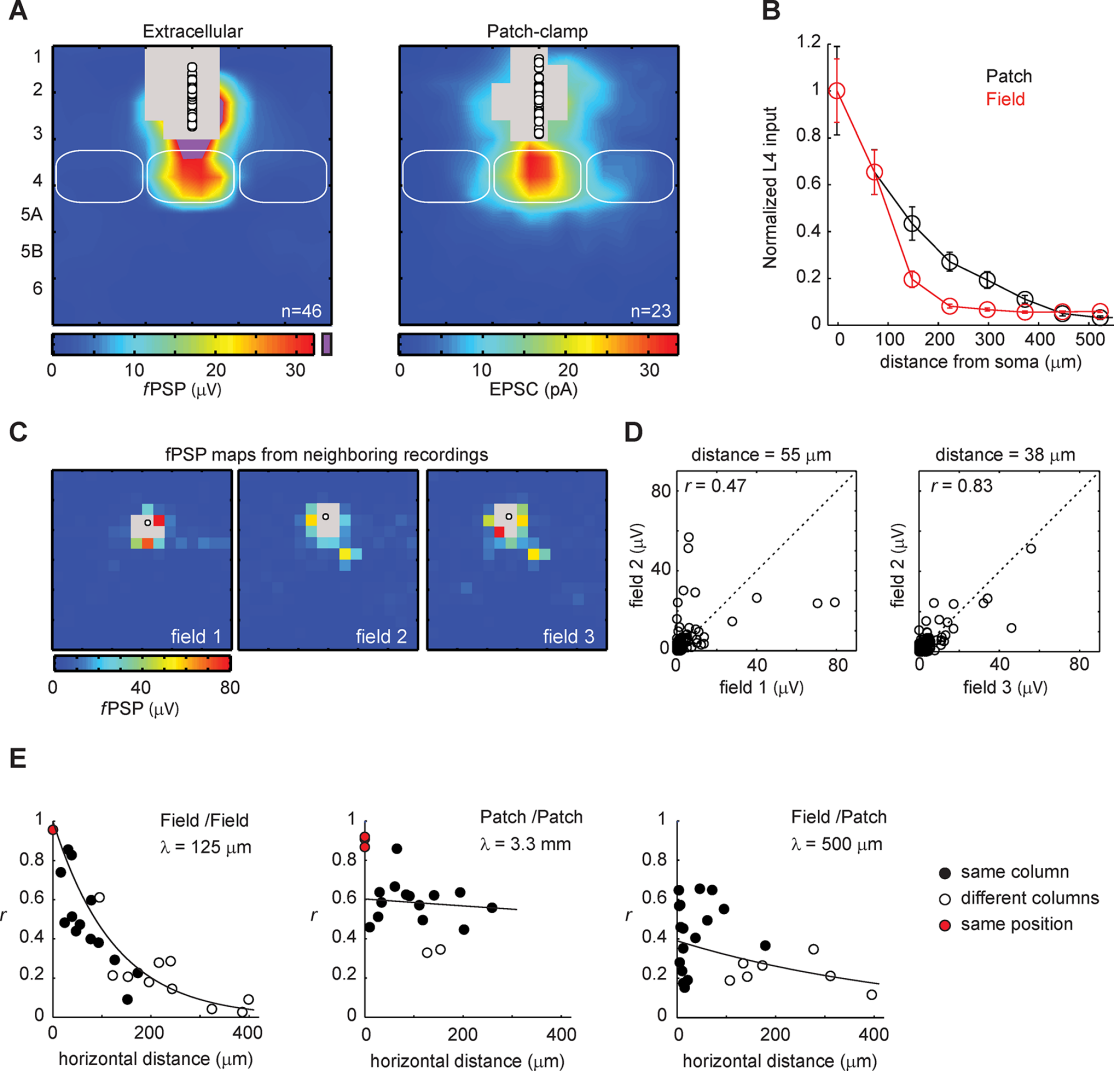
Comparison of the fPSP maps with maps computed from excitatory postsynaptic currents recorded in patch-clamp. **A,** The group-average fPSP map (left) and EPSC map (right) computed from recordings in layer 2/3 show similar patterns of inputs. The region in purple is where fPSP peak amplitudes were greater than the upper limit of the color scale. Numbers on the side indicate the layers. The regions in grey are where directly-evoked responses prevented the measurement of synaptic responses. **B**, Horizontal profile of synaptic inputs evoked by stimulations in layer 4 and received by layer 2/3 for extracellular field recordings (red) and patch-clamp recordings (black).**C**, Three fPSP maps obtained from a single slice. **D**, The plots show the pixel-wise comparison for two pairs of maps shown in C. *r* is the correlation coefficient. The diagonal dashed line shows the perfect correlation. Some sites gave inputs in one of the maps only, yielding points that fall along the zero lines (horizontal/vertical axis). The distances indicated above the plots are the horizontal distances between recording positions. **E**, Correlation coefficient as a function of distance between recording positions in pairwise comparisons of fPSP maps (left) or EPSC maps (middle). The right panel shows the comparison between fPSP and EPSC maps. The black symbols are for pairs recorded in a same cortical column, the whites for pairs recorded in neighboring columns and the red for pairs recorded at the exact same position (i.e., repetitions).

We next directly compared fPSP maps and EPSC maps generated from a same layer. L2/3 cells recorded in patch-clamp were held at -70 mV, close to the reversal potential of inhibitory currents, in order to isolate EPSC. We found that the origin of synaptic inputs was more spatially diffuse in the EPSC maps than in the fPSP maps (Fig 3A and 3B). The distance along the horizontal axis between the recording site and the origin of L4 input was 87 ± 9 μm in EPSC maps (n = 23) and 24 ± 3 μm in fPSP maps (n = 46, p < 0.001; see Methods).

This dissimilarity along the horizontal axis between fPSP maps and EPSC maps was evaluated further with pairwise comparisons. Recordings in single cell voltage clamp mode and/or in extracellular mode were performed in L3 either simultaneously or sequentially on the same slice keeping the coordinates of the uncaging grid identical (see Methods). To quantify similarities and differences in pairs of input maps, we computed correlation coefficients on a pixel-by-pixel basis (Fig 3C and 3D).

We first tested whether the two recording methods yielded the same reliability and single-position maps were generated twice for a subset of experiments. Correlation coefficients were high both in extracellular mode and in single-cell voltage-clamp (field, 0.95 ± 0.001, n = 2; patch, 0.90 ± 0.02, n = 3; Fig 3E) indicating that responses evoked over the course of several maps (8 total) were stable for both recording methods.

We next evaluated the map correlations for pairs of cells separated by various horizontal distances. It was previously shown for rat barrel cortex that EPSC maps showed similar patterns if they were from the same barrel-column, independently of the intersomatic distance of the cells: map correlation was stable over the extent of a barrel, consistent with the importance of the column-unit in the anatomy of cortical networks [27]. We repeated these measurements for our extracellular and patch-clamp recordings in mice and compared the distributions of correlation coefficients as a function of inter-pipette distance (Fig 3E). We used a combination of simultaneous and sequential mapping to record input maps for groups of two to four recording sites per slice and we performed all possible pairwise comparisons and computed correlation coefficient for each.

Like in rats, intersomatic distance was a poor predictor of correlation coefficient (*r*) for EPSC maps: *r* was on average 0.59 ± 0.03 (from 0.45 to 0.86, n = 14 pairs; Fig 3E) for cells located in a same column and showed almost no decline for distances up to 260 μm. However, correlation dropped if cells were in different columns (*r* = 0.34 ± 0.01, n = 2).

In contrast, correlation of fPSP maps showed a steep decline for increasing distances, regardless of whether recordings were in a same column or not: *r* started higher between 0.8 and 0.9 for distances shorter than 40 μm but was already down to 0.5 at 80 μm and down to 0.2 at 200 μm (n = 27 pairs; Fig 3E). The distribution could be fitted with an exponential and yielded a length constant (λ) of 125 μm. In contrast, λ was 3.3 mm for patch-clamp recordings.

The distribution of correlation coefficients for 'field/patch' pairwise comparisons had attributes from the two distributions described above (n = 24 pairs; Fig 3E): Like for patch-clamp, correlation started low with *r* close to 0.4 for small inter-pipette distances. In addition, similar to field recordings, *r* decreased rapidly as the inter-pipette distance increased regardless of the column boundaries. The distribution fitted with an exponential yielded a λ of 500 μm.

**Fig 4:**
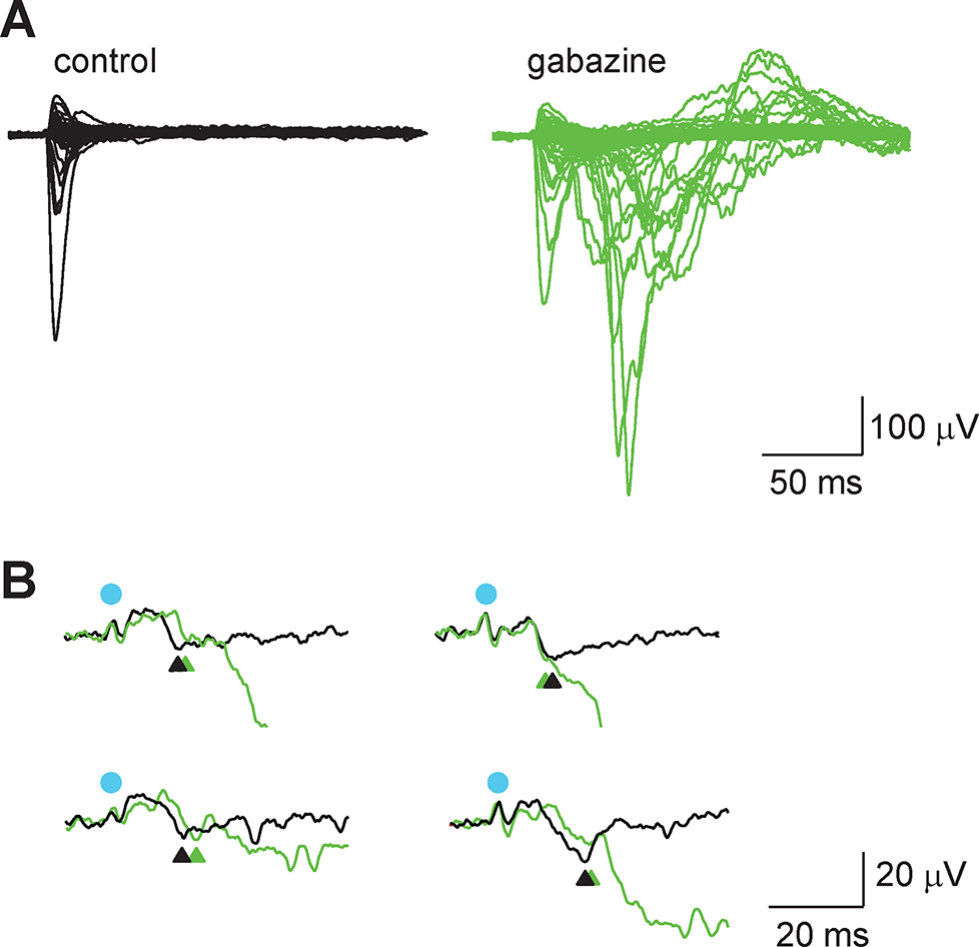
The block of GABA_A_ receptors has little effect on fPSP postsynaptically. **A**, Superposition of the 256 traces of a map recorded in layer 3 in control condition (black) and in the presence of gabazine (green). Blocking the synaptic inhibition increased and prolonged considerably the excitation of neurons stimulated by the uncaging of glutamate. **B**, Superposition of responses evoked by stimulating in layer 4 and recorded in control and in the presence of gabazine. The blue dots indicate the stimulation and the arrow heads the synaptic events chosen for the comparison of their slope.

Because glutamate-uncaging excites both excitatory and inhibitory neurons, we next evaluated the respective bearing of excitation and inhibition on the extracellular currents by testing the effects of gabazine (5 mM), a blocker of GABA_A_ receptors. Could synaptic inhibition be responsible for the sharp spatial profile of L4 inputs in the fPSP maps? Experiments were performed in the presence of high extracellular Mg^2+^ in an attempt to contain the enhancement of network activity that was bound to happen once inhibition would be silenced in the slice. Raising Mg^2+^ to 6 mM (Ca^2+^ was at 4 mM) failed to prevent this phenomenon completely because LFP traces showed the signs of a larger, prolonged and most likely indirect excitation of presynaptic neurons (Fig 4A). This prevented a simple comparison of the fPSP maps with and without gabazine. However, it was possible at some sites to separate an early component of the evoked response from the delayed polysynaptic events that were induced in the presence of gabazine (Fig 4B). On average, gabazine decreased the slope of this early fPSP evoked from L4 stimulations by—6 ± 4% (n = 74 measurements over 4 trials; p < 0.05). This suggests that although blocking inhibition has a dramatic effect on the excitation of L4 cells, the contribution of GABAergic transmission to the size of fPSP is small. Hence, it is unlikely that synaptic inhibition explains the sharp spatial profile of fPSP seen in Fig 3A and the loss of the column-unit in pairwise comparisons (Fig 3E).

**Fig 5:**
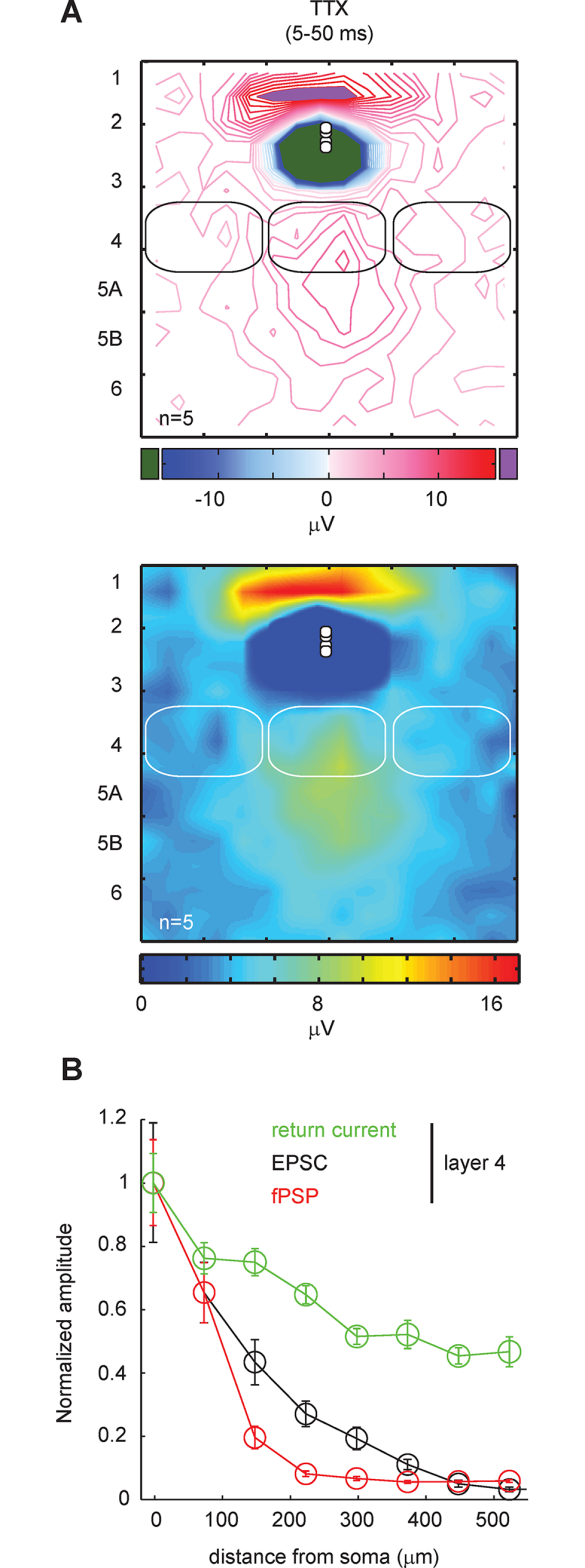
Return currents of direct depolarizations alter the pattern of synaptic inputs in the fPSP maps. **A**, Top panel, an averaged isoline map showing the mean amplitude of direct evoked potentials measured in the synaptic window (5–50 ms post stimulus onset) in the presence of TTX. The red isolines show the positive potentials, the blue the negative potentials. Return currents (in red) were still detected in the synaptic window when stimulating in the layers 4 and 5. Bottom panel, the same positive return currents shown in a color map. Recordings (n = 5) were in layer 3. **B**, Horizontal profile of return currents evoked by stimulations in the layer 4 in the presence of TTX (green) superposed on the horizontal profiles of fPSP (red) and EPSC (black) evoked by stimulations in layer 4.

We previously described how LSPS evoked direct responses under the form of return currents when glutamate was uncaged above and below the recording site (Fig 1B–1D). Because return currents had a slow kinetic and were partially masking the responses arising in the synaptic window, we investigated whether they could be responsible for the sharp horizontal profile of L4 synaptic inputs in the fPSP map generated from L2/3 recordings (Fig 3B). Fig 5A shows the map of direct responses evoked in the presence of TTX (n = 5) and Fig 5B their horizontal profile in the L4 region. The amplitude of return currents measured in the synaptic window (5–50 ms post stimulus) was the largest at stimulation sites that were vertically aligned with the recording pipette and decreased slowly as a function of distance. The slope to reach a minimum was shallower than in the horizontal profile obtained for EPSC (Fig 5B) and residual positive potentials were still visible at the edges of the LSPS map. Hence, it is likely that glutamate-evoked direct responses alter the shape of L4 input in the fPSP map and that their interference is the most detrimental at stimulation sites that are off-centered with the recording pipette where synaptic responses are on average smaller and return currents still relatively large.

## Discussion

We find that local field potentials can be evoked by LSPS with glutamate-uncaging in barrel cortex in similar experimental conditions that were previously used for LSPS combined with patch-clamp recordings. Maps computed from the peak measurements of evoked synaptic negative potentials revealed some well known features of cortical excitatory networks: a layer-specific pattern of inputs and a vertical organization of projections.

Although LFP are simple to record, their interpretation is more complex. One same event can take two appearances, either positive or negative local field potential, depending on the distance between the recording site and where the event is taking place (reviewed in [29]). However, by focusing on negative potentials, we selected the depolarizing events that occurred in the vicinity of the pipette tip, most likely in cells whose soma was located nearby. This was made particularly clear in the case of direct glutamate-evoked responses: All responses arose at the stimulus onset and were TTX resistant, but they were of two types: the first type was a large negative potential evoked when glutamate was uncaged at the pipette tip. These depolarizations were analogous to the direct AMPA currents that were observed in LSPS maps computed from patch-clamp recordings and they occupied a small region in the map. The second type was a smaller positive potential when glutamate was uncaged at greater distances from the pipette tip. These responses were return currents of depolarizations that occurred in distal dendrites of the recorded cells or in the soma of neurons located in a different layer. They occupied a large portion of the LSPS maps. Based on these observations, we decided to generate functional connectivity maps based on the evoked synaptic negativities so as to constrain as much as possible the detection to synaptic events that occurred in cells with a soma located near the pipette tip.

The fPSP maps have hallmarks of connectivity maps generated with LSPS: 1- The stimulation sites yielding synaptic inputs are distributed in small hotspots, down to 1 pixel in size, in an individual map. 2- The fPSP maps are highly reproducible across repeated measurements. 3- They are highly variable when generated for different recording sites, provided that a minimum distance of 40 μm is respected. 4- The group-average maps show distinct patterns of inputs for each layer.

Moreover, the resemblance of the fPSP maps with the EPSC maps is striking. This is true for the recordings performed in L2/3 (Fig 3A) as well as for every other layer. Indeed, the six maps in Fig 2 show the major ascending and descending pathways that were previously described for cells recorded in the voltage-clamp mode at -70 mV: L2 and L3 received major inputs from L4 [9,23], while L5A and L5B received theirs from L2/3 [7,9,33]. The subtle differences between laminae were visible too. For instance, cells in L5B received inputs from a lengthy region in L2/3 whereas L5A cells were targeted by superficial L2/3 neurons only [9]. One other detail is that L4 cells targeted L3 more strongly than L2 [1,9,23,34]. This suggests that fPSP maps not only permit a qualitative description of the intracortical functional connectivity, they also deliver quantitative information. One marked difference between the fPSP and EPSC maps is the lack of L5A input targeting L2 [9,23,34]. The probability of finding a L5A pyramidal cell connected to a L2 pyramidal cell is low in barrel cortex, ~ 4%, which is about two times lower than finding the reverse connection and three times lower than finding a L4 to L3 connection [1]. A weak L5A to L2 projection might also have been masked by return currents (Fig 5A) or by noise in the extracellular recordings. The comparison of the 2009 study from Lefort and collaborators with this one suggests that fPSP maps display connections with probability > 5%. Unexpectedly, a connection from L5A to L4 was observed in fPSP maps whereas it was especially rare (< 1%) in previous studies [1,9]. Our preliminary unpublished work investigating L4 EPSC maps suggests that the age of the animals which were two to three weeks older here could explain this discrepancy. Finally, as in EPSC maps [9], the fPSP maps showed that L6 neurons were principally connected to themselves. However, L4 to L6 excitatory synaptic connections were observed in dual patch-clamp recordings [35]. The fact that synapses between L4 spiny stellate cells and L6 neurons are principally located on the distal dendrites of L6 neurons within the layer 4 [35] could explain why they were not detected by positioning extracellular electrodes in L6. This exception highlights one property that is true for most interlaminar excitatory networks within barrel cortex and that helps making the fPSP maps and EPSC maps so similar: their synapses are the densest on dendrites close to the cell body of the postsynaptic cells [36,37,38]. Hence, fPSP are more likely to originate in cells whose cell-body is located near the tip of the recording electrode.

Our data suggests that GABAergic transmission has little bearing on the size of fPSP. We found that gabazine's major if not only effect was to unleash the excitation of presynaptic neurons. This lack of effect on the postsynaptic side was expected given that responses became globally more positive in the presence of TTX (Fig 5), which suggested that positive potentials measured in the synaptic window were principally the residuals of direct responses. The lack of obvious contribution of GABA differs from other studies where synaptic inhibition contributes actively to shaping LFP (reviewed in [29]). One may attribute this specificity to a combination of factors linked to the stimulation method and to the recording mode. With LSPS, neurons are excited at the level of their cell body not their axon and the propagation of input is blocked after the very first synapse (i.e. transmission is monosynaptic only, [4]). Hence, the effective stimulation is confined to a small volume (~ 80 μm diameter, < 100 μm in depth; ~ 45 neurons in the L4 and L2/3; [23]) which is the condition for interpreting the LSPS map. This stimulation method gives an advantage to the excitatory projections because of the prevalence of glutamatergic neurons in cortex (70–90% in L2-6; [39]). Despite this, large inhibitory postsynaptic currents are evoked with LSPS and inhibitory maps have been generated when neurons were recorded in patch-clamp at a depolarized potential [6]. The advantage given to the excitatory connections is reinforced in the present study by the fact that responses are recorded from the extracellular space. Indeed, multiple currents must overlap in time in order to be detected [29]. This implies that several presynaptic–connected–neurons must be activated simultaneously for a projection to emerge in the fPSP maps. This is more likely to happen for the glutamatergic neurons given their high density at any given stimulated site. The effect of gabazine was evaluated on the synaptic events evoked by stimulations in L4. It is possible that the blocker had a pronounced effect on fPSP evoked at sites overlapping with the recorded neurons in L3 because this is where the density of presynaptic interneurons was the highest [6]. However, synaptic responses could not be disambiguated from the evoked direct responses at these sites.

Despite their similarities, the average fPSP map and EPSC map generated from recordings in L2/3 showed an important difference when the spatial distribution of inputs was compared along the horizontal axis: inputs were narrowly distributed in the fPSP map, and the averaged hotspot was vertically aligned with the recording site. As a direct consequence, pairwise comparison of fPSP maps did not bring to light the column-unit in the organization of excitatory projections because sites with inputs trailed the recording location closely. There is likely more than one reason for this phenomenon. First, direct return currents partially masked the synaptic responses and their size declined with increasing distances at a slower pace than the size of synaptic responses. This means that fPSP maps must be interpreted in the scope of the spatial distribution of directly evoked return currents. A second factor that could control the spatial distribution of fPSP in the map is the anatomy of the L4 to L2/3 projections. Indeed, L4 neurons project their axons in L2/3 in an almost straight vertical line. Their morphological reconstruction shows that the half-width of their distribution is 100–150 μm on each side of the cell body [17,40,41]. This value is also the half-width of the distribution of L4 inputs in the fPSP map (Fig 3B). Hence, the stimulation at sites vertically aligned with the recording pipette excites the largest number of presynaptic L4 cells and it is also the most efficient at activating a large number of synapses located in the vicinity of the recording pipette tip.

To conclude, we propose that LSPS combined with field recording is a simple and sensitive assay to quickly map all the excitatory projections that are not severed in a slice. One could increase the yield per animal further by increasing the recording capacity with multi-electrode probes [25]. This would also permit current source density analysis. The approach should help providing a connectivity diagram to cortical areas less explored than barrel cortex. It could also drive the way research on the effects of a genotype or any other manipulation is planned. Indeed, the decision to focus in-depth investigations of cellular mechanisms on a given connection could be based on a first round of empirical observations aimed at selecting the largest or most interesting difference and not on an initial bias. Finally, investigating the transformation of several projections in same animals as opposed to investigating them serially could bring up correlated changes and allow extracting knowledge from inter-individual variability.
